# Factors associated with exclusive breastfeeding among infants under six months of age in peninsular malaysia

**DOI:** 10.1186/1746-4358-6-2

**Published:** 2011-02-02

**Authors:** Kok Leong Tan

**Affiliations:** 1 Community Medicine Division, International Medical University, Bukit Jalil, 57000 Kuala Lumpur, Malaysia

## Abstract

**Background:**

Breastfeeding is accepted as the natural form of infant feeding. For mothers to be able to breastfeed exclusively to the recommended six months, it is important to understand the factors that influence exclusive breastfeeding. The aim of the study was to identify factors associated with exclusive breastfeeding in Peninsular Malaysia.

**Methods:**

This was a cross-sectional study involving 682 mother-infant pairs with infants up to six months attending maternal and child health section of the government health clinics in Klang, Malaysia. Data were collected by face-to-face interviews using a pre-tested structured questionnaire over 4 months in 2006. Data on breastfeeding were based on practice in the previous *one month *period. Logistic regression was used to assess the independent association between the independent variables and exclusive breastfeeding adjusting for infant age.

**Results:**

The prevalence of exclusive breastfeeding among mothers with infants aged between one and six months was 43.1% (95% CI: 39.4, 46.8). In the multivariate model exclusive breastfeeding was positively associated with rural residence, Malay mothers, non-working and non-smoking mothers, multiparous mothers, term infants, mothers with husbands who support breastfeeding and mothers who practice bed-sharing.

**Conclusions:**

Interventions that seek to increase exclusive breastfeeding should focus on women who are at risk of early discontinuation of breastfeeding.

## Background

Over the past decade, the government of Malaysia has recognized the significance of breastfeeding and infant nutrition. The National Breastfeeding Policy was formulated in 1993 and revised in 2005 in accordance with the World Health Assembly Resolution 54.2 (2001) whereby exclusive breastfeeding was recommended for the first six months of life as a public health measure and thereafter continued up to two years of age and beyond with timely, adequate and safe complementary foods [1]. According to Malaysia Third National Health and Morbidity Survey 2006 (NHMS III), the prevalence of infants who initiated breastfeeding within one hour of birth was 63.7% (95% CI: 61.4, 65.9) while the prevalence among children less than 12 years ever breastfed was 94.7% (95% CI: 93.0, 95.9) [2]. The prevalence of exclusive breastfeeding up to four months and six months were 19.3% (95% CI: 15.5, 23.9) and 14.5% (95% CI: 11.7, 17.9) respectively [2].

Wide variations exist in breastfeeding and other infant feeding practices between countries and among subgroups of populations. For mothers to be able to breastfeed exclusively to the recommended six months, it is important to understand the factors that influence exclusive breastfeeding. Various factors have been found to be associated between exclusive breastfeeding and breastfeeding initiation and duration; socio-demographic factors (education level, urban versus rural residence, monthly household income and parity); biosocial factors (breastfeeding support); cultural factors (beliefs, norms and attitudes towards breastfeeding) and employment policies [3-7].

There is limited published data regarding exclusive breastfeeding determinants in Malaysia. This study was conducted to identify determinants of exclusive breastfeeding in a group of mother-infant pairs attending maternal and child health section of the government health clinics in Klang, Malaysia.

## Methods

This was a cross-sectional study conducted in Klang, Malaysia involving 682 mother-infant pairs with infants up to six months attending the Maternal and Child Health (MCH) section of the government health clinics over four months in 2006. An analysis of bed-sharing practices has already been published [8]. In Klang, there are eight government health clinics with their subsidiary community clinics providing health needs for the community. Two of the health clinics including their subsidiary community clinics were chosen for the study by random selection where one health clinic (Bukit Kuda Health Clinic) serves an urban population while the other (Kapar Health Clinic) serves a rural population.

The sampling method used was universal sampling. All eligible mother-infant pairs who attended the clinics during the study period were included in the study. Data collection was carried out according to a schedule that was prepared. Data was collected from the two identified areas in the study at alternate weeks with designated days for each clinic. The specific days selected for data collection for each clinic coincided with the days when immunization was scheduled for the infants.

The inclusion criteria in the study included all mother-infant pairs visiting Bukit Kuda and Kapar Health Clinic including the subsidiary community clinics between 19 June and 19 October 2006. Mother-infant pairs were excluded from the study if mothers were less than 18 years old, have a child with congenital malformation, infants more than six months old, infant taken care of by caregivers during night time and infant not accompanied by mother at the clinic.

After consent from eligible mothers, a face-to-face interview using a pre-tested pre-coded structured questionnaire was conducted by the author in the clinics at respective days during the study period. The questionnaire was pre-tested among 35 mother-infant pairs from another health clinic in the same district. Data regarding maternal socio-demographic variables (area of residence, ethnicity, age, body mass index, marital status, education level, occupation, monthly household income and smoking status), paternal socio-demographic variables (ethnicity, age, education level, occupation, smoking status), biomedical variables (parity, number of antenatal visits, mode of delivery, infant gender, infant gestational age, infant birth weight, breastfeeding class attendance), household variables (number of people staying in the house, number of rooms in the house, number of children under 12 years staying in the house and occupancy rate) and psychosocial variables (husbands' support toward breastfeeding, bed-sharing practice) were collected. The interview was conducted in 'Bahasa Malaysia' as it was the national language and widely used by the respondents. For mothers of Chinese ethnicity not well versed in 'Bahasa Malaysia', the interview was conducted in Mandarin language. In order to ensure no eligible mother-infant pairs were missed from the interview, the author attended the clinics at least fifteen minutes before the immunization session started. Liaison with the nurses at the clinics was also made to ensure that all eligible mother-infant pairs were interviewed. Socio-demographic data were also collected from all eligible mother-infant pairs who refused to participate in the study. A yellow sticker was placed on the right upper corner of the infants' immunization card after the interview to prevent interviewing the same mother-infant pair during the entire four months of data collection.

In this study, exclusive breastfeeding was defined as the infant having received only breast milk from the mother (either directly from the breast or expressed) and no other liquids or solids with the exception of drops or syrups consisting of vitamins, mineral supplements, or medicines over the *one month *period prior to the interview. A recall over a one month period was used in this study because this duration coincided with the immunization days where infants were called in every month for the first six months of life.

Bed-sharing was defined as an infant sharing a bed with mother within arm's reach of the mother. A bed was defined as either a sleeping mattress placed on a bed frame or a sleeping mattress placed on the floor. Mothers were defined as a 'bed-sharer' if they shared a bed with their infant for three or more times a week for all or part of the night.

Occupation was defined as the working status of the mother. Working encompasses any form of employment with contribution to income tax.

All data in the questionnaire were coded and entered into Statistical Package for the Social Sciences (SPSS) version 11.0. The dependent variable was exclusive breastfeeding. Univariate associations between various factors and exclusive breastfeeding adjusting for infant age were identified and multiple logistic regression was used to construct the model to examine the independent association of various factors to exclusive breastfeeding while simultaneously controlling for potential confounders. The statistical test used was binary logistic regression and the level of significance was set at p < 0.05. Odds ratio (OR) and 95% Confidence Interval (CI) were calculated as a measurement of association between an independent variable and the outcome. All variables associated with exclusive breastfeeding in the univariate analyses were included in the initial multivariate model. Variables were excluded from the final logistic model if they were not associated with exclusive breastfeeding and their removal from the model did not materially affect the association of other variables in the model based on the algorithm proposed by Hosmer and Lemeshow [9].

The project received ethical approval from the University Malaya Medical Faculty Ethics Committee and Medical Research Committee Ministry of Health Malaysia. Signed informed consent was obtained from all the participants. Confidentiality of the data and the privacy of mothers were respected at all times.

## Results

Among 712 eligible mother-infant pairs during the study period, 682 respondents were included in the study giving a response rate of 95.8%.

Table 1 showed the socio-demographic characteristics among the respondents. The mean (SD) maternal age was 28.4 (5.1) years. Among those interviewed, 59.7% of the mother-infant pairs were from urban area while 40.3% were from rural area. Malays formed the largest ethnic group (60.9%) followed by Chinese, Indian and other ethnic groups (22 Indonesian, 5 Thailand, 2 Burmese and 1 Caucasian). The majority of the mothers (78.1%) had secondary school level education, were not working (57.6%) and from a monthly household income of between RM1,500 and RM3,500 (66.1%). Maternal Body Mass Index (BMI) ranged between 15.0 and 47.9 kg/m2. Almost two thirds of the mothers were multiparous (63.8%). The majority of mothers gave birth by normal vaginal delivery (84.9%) and with normal birth weight of between 2,500 g and 4,000 g (85.2%). There were slightly more male infants (53.7%) than female infants (46.3%); the age of infants at interview ranged from one to six months.

**Table 1:** The socio-demographic characteristics of respondents (n = 682)

Characteristics	n	%
**Maternal age (years)**		
< 25	140	20.5
25 - 34	445	65.3
34 - 43	97	14.2
**Area of residence**		
Urban	407	59.7
Rural	275	40.3
**Maternal ethnicity**		
Malay	415	60.9
Chinese	128	18.7
Indian	109	16.0
Other	30	4.4
**Maternal education level**		
Diploma/Degree	109	16.0
Secondary school	533	78.1
Primary school	40	5.9
**Maternal occupation**		
Working	289	42.4
Not working	393	57.6
**Maternal BMI (kg/m^2^)**		
<18.5	41	6.0
18.5 - 24.9	446	65.4
>24.9	195	28.6
**Monthly household income (RM)**		
>3,500	52	7.6
1,500 - 3,500	451	66.1
<1,500	179	26.3
**Parity**		
Primiparous	247	36.2
Multiparous	435	63.8
**Mode of delivery**		
Vaginal	579	84.9
Instrumental/LSCS	103	15.1
**Infant gender**		
Male	366	53.7
Female	316	46.3
**Infant birth weight (g)**		
<2,500	86	12.6
2,500 - 4,000	581	85.2
>4,000	15	2.2
**Infant age (months)**		
One	139	20.3
Two	111	16.3
Three	123	18.0
Four	96	14.1
Five	102	15.0
Six	111	16.3

From this study, the prevalence of exclusive breastfeeding among mothers with infants aged between one and six months was 43.1% (95% CI: 39.4, 46.8). The prevalence of exclusive breastfeeding when stratified by infant age from one to six months ranged between 32.4% and 63.3% with the highest among one month old infants and lowest among six month old infants (Table 2).

**Table 2:** Prevalence (95% CI) of exclusive breastfeeding by infant age

Characteristics	Prevalence (95% CI) of exclusive breastfeeding
**Infant age (months)**	
One	63.3 (55.2, 71.4)
Two	43.2 (33.9, 52.6)
Three	36.6 (28.0, 45.2)
Four	40.6 (30.6, 50.6)
Five	37.3 (27.7, 46.8)
Six	32.4 (23.6, 41.3)

Infant age (one to six months)*	43.1 (39.4, 46.8)

* the prevalence is age-standardised

The following were associated with exclusive breastfeeding in bivariate analysis adjusting for infant age: area of residence, maternal ethnicity, education level, occupation, monthly household income, maternal smoking status, parity, infant gestational age, husbands support on breastfeeding, number of children under 12 years saying in the house, occupancy rate and bed-sharing practice (Table 3).

**Table 3:** Logistic regression of determinants of exclusive breastfeeding

Variables	Exclusive breastfeeding (n = 294) n (%)	Non-exclusive breastfeeding (n = 388) n (%)	Age-adjusted		Multivariate	
			
			OR	95% CI	Adj OR	95% CI
**Area of residence**						
Urban	167 (41.0)	240 (59.0)	1.00	-	1.00	-
Rural	127 (46.2)	148 (53.8)	1.23	1.08, 1.68	1.16	1.03, 1.89
**Maternal ethnicity**						
Malay	218 (52.5)	197 (47.5)	1.00	-	1.00	-
Chinese	20 (15.6)	108 (84.4)	0.17	0.10, 0.28	0.20	0.11, 0.35
Indian	39 (35.8)	70 (64.2)	0.50	0.33, 0.78	0.05	0.29, 0.75
Other	17 (56.7)	13 (43.3)	1.18	0.56, 2.50	1.00	0.45, 2.26
**Maternal education level**						
Diploma/Degree	37 (33.9)	72 (66.1)	1.00	-	n/s	
Secondary school	237 (44.5)	296 (55.5)	1.56	1.01, 2.40		
Primary school	20 (50.0)	20 (50.0)	1.95	1.17, 4.06		
**Maternal occupation**						
Working	73 (25.3)	216 (74.7)	1.00	-	1.00	-
Not working	221 (56.2)	172 (43.8)	3.80	2.73, 5.30	3.66	2.45, 5.46
**Monthly household income (RM)**						
>3,500	5 (9.6)	47 (90.4)	1.00	-	n/s	
1,500 - 3,500	188 (41.7)	263 (58.3)	6.68	2.61, 17.08		
<1,500	101 (56.4)	78 (43.6)	12.10	4.61, 31.80		
**Maternal smoking status**						
Yes	1 (5.3)	18 (94.7)	1.00	-	1.00	-
No	293 (44.2)	370 (55.8)	14.24	1.89, 107.16	5.18	1.59, 45.05
**Parity**						
Primiparous	81 (32.8)	166 (67.2)	1.00	-	1.00	-
Multiparous	213 (49.0)	222 (51.0)	1.97	1.42, 2.72	1.68	1.17, 2.42
**Infant gestational age (weeks)**						
<37	20 (29.4)	48 (70.6)	1.00	-	1.00	-
≥37	274 (44.6)	340 (55.4)	1.93	1.12, 3.34	1.74	1.05, 3.01
**Husbands support on breastfeeding**						
No	3 (10.0)	27 (90.0)	1.00	-	1.00	-
Yes	291 (44.6)	361 (55.4)	7.22	2.17, 23.99	4.20	1.12, 15.75
**No children < 12 staying in house**						
1	73 (34.4)	139 (65.6)	1.00	-	n/s	
2 - 4	193 (46.3)	224 (53.7)	1.64	1.16, 2.31		
>4	28 (52.8)	25 (47.2)	2.13	1.16, 3.92		
**Occupancy rate (person per room)**						
<1.5	46 (32.6)	95 (67.4)	1.00	-	n/s	
1.5 - 4.0	238 (45.1)	290 (54.9)	1.69	1.15, 2.51		
>4.0	10 (76.9)	3 (23.1)	6.86	1.80, 26.08		
**Bed-sharing practice**						
No	45 (24.9)	136 (75.1)	1.00	-	1.00	-
Yes	249 (49.7)	252 (50.3)	2.99	2.04, 4.37	1.50	1.12, 2.37

OR: odds ratio; CI: confidence interval; n/s: not significant

Adj OR: adjusted odds ratio; adjusted for area of residence, maternal ethnicity, maternal occupation, maternal smoking status, parity, infant gestational age, husbands support on breastfeeding, bed-sharing practice and infant age.

All the variables associated with exclusive breastfeeding in the infant age-adjusted analyses were included in a multivariate model. Variables that were significantly associated with exclusive breastfeeding include area of residence, maternal ethnicity, maternal occupation, maternal smoking status, parity, infant gestational age, husbands support on breastfeeding and bed-sharing practice (Table 3). Interaction test was performed among the variables in the multivariate final model which showed no interactions. Mothers from rural area were more likely to exclusively breastfeed compared to mothers from urban area (OR = 1.16; 95% CI: 1.03, 1.89). Chinese mothers were 0.12 times less likely to exclusively breastfeed compared to Malay mothers (OR = 0.20; 95% CI: 0.11, 0.35). Non-working mothers were 3.5 times more likely to exclusively breastfeed compared to working mothers (OR = 3.66; 95% CI: 2.45, 5.46). Non-smoking mothers were five times more likely to exclusively breastfeed compared to smoking mothers (OR = 5.18; 95% CI: 1.59, 45.05) while multiparous mothers were almost twice more likely to exclusively breastfeed compared to primiparous mothers (OR = 1.68; 95% CI: 1.17, 2.42). Mothers with term infants (≥ 37 weeks gestation) were twice as likely to exclusively breastfeed compared to mothers with preterm infants (< 37 weeks gestation) (OR = 1,74; 95% CI: 1.05, 3.01). Mothers with supportive husbands on breastfeeding were four times more likely to exclusively breastfeed compared to non-supportive husbands (OR = 4.20; 95% CI: 1.12, 15.75). Mothers who practiced bed-sharing with their infants were 1.5 times more likely to exclusively breastfeed compared to mothers who did not practice bed-sharing (OR = 1.50; 95% CI: 1.12, 2.37).

## Discussion

Adequate nutrition is critical for child health and development. The period from birth to two years of age is particularly important because of the rapid growth and brain development [10,11]. The period is often marked by growth faltering, micronutrient deficiencies and common childhood illnesses such as diarrhoeal diseases, as a child is introduced to solid foods in addition to breast milk [12-14].

In this study, the overall prevalence of exclusive breastfeeding among mothers with infants up to six months of age was 43.1%. This result was higher compared to the national figure [2]. Other studies reported prevalence of exclusive breastfeeding between 12.5% and 48% [15-17]. Under the Malaysia National Study, a 24 hour recall period was used. The WHO has developed a set of definitions and indicators of infant feeding patterns that can be applied in assessing breastfeeding practices in household surveys using the 24 hour recall methodology [18]. In this study, data on breastfeeding was based on feeding practice over a period of one month prior to the interview which is a non-standard practice to collect breastfeeding data. The validity of data on exclusive breastfeeding based on 24 hour periods has also been questioned [19]. Aarts et al. reported a wide discrepancy on the prevalence of exclusive breastfeeding between current status based on a 24-hour recording and exclusive breastfeeding since birth [20]. Several factors may have led the study to overestimate the prevalence of exclusive breastfeeding of the population. Since the interview was conducted face-to-face, by the author who is a medical doctor, in the health clinics which strongly promote exclusive breastfeeding, information bias is possible. A recall period of one month may lead to recall bias among respondents. The majority of the respondents were Malays, of low family income and not working. Selection bias may have occurred as studies have showed that these factors were strongly associated with exclusive breastfeeding in Malaysia [2,15-17].

This study identified seven factors associated with exclusive breastfeeding. Mothers from rural area more commonly exclusively breastfeed compared to mothers from urban area which was supported by many articles and reports [2,3,7]. Chinese mothers exclusively breastfeed the least among the ethnic groups in the study. In Malaysia, it is customary for Chinese mothers to employ an experienced helper to take care of the infant and the household needs during the first month of confinement. This situation leads to reduced effort and opportunity for breastfeeding. Non-working mothers were positively associated with exclusive breastfeeding. This association has been reported by Senarath et al [21] for Timor-Leste, Ong et al [6] and Chen [22] for Singapore. This result does not necessarily mean that working leads to failure to exclusively breastfeed. Additional factors such as weaning in preparation to return to work, maternal fatigue and the difficulty in juggling the demands of work and breastfeeding may also contribute to this issue. In Malaysia, working mothers are given only two months maternity leave and facilities for breastfeeding at work places are not acceptable or flexible. This situation would deter working mothers from exclusively breastfeeding as compared to housewives [23].

Non-smoking mothers were more likely to breastfeed than mothers who smoked in this study as in other studies [24,25]. This study found that exclusive breastfeeding was more common among mothers with more than one child. Studies conducted in Malaysia and Hong Kong reported similar findings where mothers with their first child were less knowledgeable and skilful in breastfeeding [26,27]. This caused low self confidence among mothers to breastfeed their infants. It is common for mother-in-laws to accompany mothers during the confinement period especially after the first delivery. Their lack of support for breastfeeding could influence mothers not to breastfeed [28].

Exclusive breastfeeding was more common among mothers with supportive husbands on breastfeeding compared to non-supportive husbands. In Malaysia or Asian setting, the husband plays a major role in decision making about family and household matters. Banks documented a highly paternalistic pattern of behaviour where husbands have traditionally held authority over many aspects of family life including intra-household decisions in an ethnographic study on family life in Kelantan, Malaysia [29]. A study by Kusago et al. concluded that among families in rural Malaysia, husbands were authoritative over intra-household decision-making [30].

In this study, bed-sharing practices appear to be positively associated with exclusive breastfeeding. A study by McKenna and colleagues concluded that routinely bed-sharing infants breastfed approximately three times longer during the night than infants who routinely slept separately which reflected a two fold increase in the number of breastfeeding episodes and 39% longer episodes [31]. Mc Coy and colleagues showed that on multivariate analysis, breastfeeding was strongly associated with bed-sharing [32]. However, since this was a cross-sectional study, the cause and effect relationship could not be established. The issue of causality - whether bed-sharing promotes breastfeeding or breastfeeding promotes bed-sharing -could not be answered.

This study has several potential limitations. The information on breastfeeding focuses within a one month period and information bias is likely the respondent. These could lead to overestimation of the prevalence of exclusive breastfeeding in this study. The cross-sectional nature of this study prevents drawing causal inferences from the association between the determinant factors and exclusive breastfeeding.

## Conclusions

Breastfeeding is the universally accepted means of infant feeding with proven benefits to the mother, infant and the economy. This study identified area of residence, maternal ethnicity, occupation, smoking status, parity, husbands support for breastfeeding and bed-sharing practice to be associated with exclusive breastfeeding. This finding applies to the target population of the study. Interventions that seek to increase exclusive breastfeeding should be more focused on women who are most at risk of early discontinuation of breastfeeding.

## Competing interests

The authors declare that they have no competing interests.
